# Isolated subdural hematoma secondary to Dural arteriovenous fistula: a case report and literature review

**DOI:** 10.1186/s12883-019-1272-z

**Published:** 2019-03-21

**Authors:** Guichen Li, Yang Zhang, Jinchuan Zhao, Xiaobo Zhu, Jinlu Yu, Kun Hou

**Affiliations:** 1 Department of Neurology, The First Hospital of Jilin University, Changchun, Jilin, China; 2 Department of Neurosurgery, The First Hospital of Jilin University, 71 Xinmin Avenue, Changchun, Jilin, 130021 China

**Keywords:** Dural arteriovenous fistula, Subdural hematoma, Cortical venous drainage, Middle meningeal artery

## Abstract

**Background:**

Dural arteriovenous fistula (DAVF) is an uncommon subtype among the intracranial arteriovenous malformations, which is characterized by pathological anastomoses between meningeal arteries and dural venous sinuses, meningeal veins, or cortical veins. While intracerebral hemorrhage accounts for most of the hemorrhagic cases in patients with DAVF, isolated subdural hematoma (SDH) is rarely reported.

**Case presentation:**

A 45-year-old man was admitted for a progressively worsening headache over 2 weeks. Head computed tomography on admission revealed an isodense chronic SDH (CSDH) on the left hemisphere with mild midline shift. Further angiography of the external carotid artery revealed a DAVF at the transverse sinus. The DAVF was embolized via the middle meningeal artery. His CSDH gradually resolved without surgical intervention. In order to further elucidate this rare entity, a review of relevant literature was also conducted.

**Conclusions:**

Isolated SDH is a rare complication of DAVF. In this report, we presented a rare case of CSDH secondary to an intracranial DAVF. According to this case report and our literature review, the so-called benign type of DAVF without cortical venous drainage does not always warrant a benign process and might be complicated with SDH. Careful preoperative investigation is needed for relative young patients presenting with idiopathic or atypical SDH.

## Background

Dural arteriovenous fistula (DAVF) is an uncommon subtype among the intracranial arteriovenous malformations (AVMs), which is characterized by pathological anastomoses between meningeal arteries and dural venous sinuses, meningeal veins, or cortical veins [1]. From a recent epidemiologic survey of DAVF in Japan, the initial clinical presentation was intracranial hemorrhage in 16% of the inflicted patients [2]. While intracerebral hemorrhage (ICH) accounts for most of the hemorrhagic cases, isolated subdural hematoma (SDH) is rarely reported [3, 4]. In the current study, we report a rare case of DAVF presenting with isolated chronic subdural hematoma (CSDH). Furthermore, a review of the literature was also conducted to further illustrate the clinical profiles of this rare entity.

## Case presentation

**Fig. 1:**
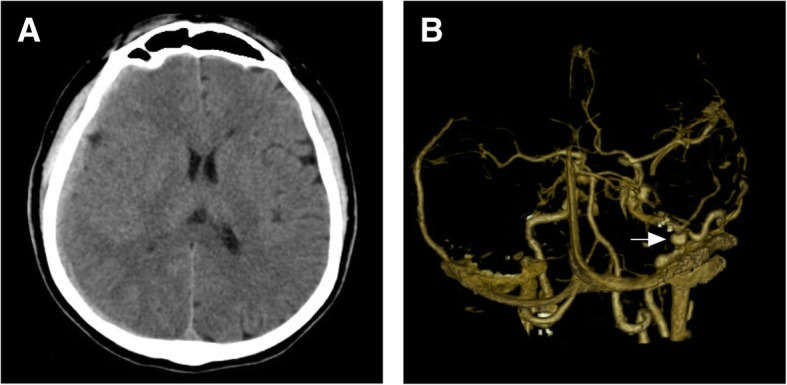
(**a**) Head CT shows an isodense CSDH on the right hemisphere with mild midline shift. (**b**) CTA reveals a DAVF located at the transverse sinus with dilated cortical venous drainage (arrow). CT: computed tomography; CSDH: chronic subdural hematoma; CTA: CT angiography; DAVF: dural arteriovenous fistula

A 45-year-old man was admitted for a progressively worsening headache over 2 weeks. He denied history of recent head trauma or anticoagulation and antiplatelet medication. General and neurologic examinations were not remarkable on admission. Routine laboratory investigations including coagulation profiles and platelet function were within normal limits. Head computed tomography (CT) on admission revealed an isodense CSDH on the right hemisphere with mild midline shift (Fig. 1a). A CT angiography (CTA) was performed to rule out any intracranial vascular malformation. A DAVF was noticed at the transverse sinus with dilated cortical venous drainage (Fig. 1b). So, a digital subtraction angiography (DSA) of the external carotid artery and DAVF embolization was planned.

**Fig. 2:**
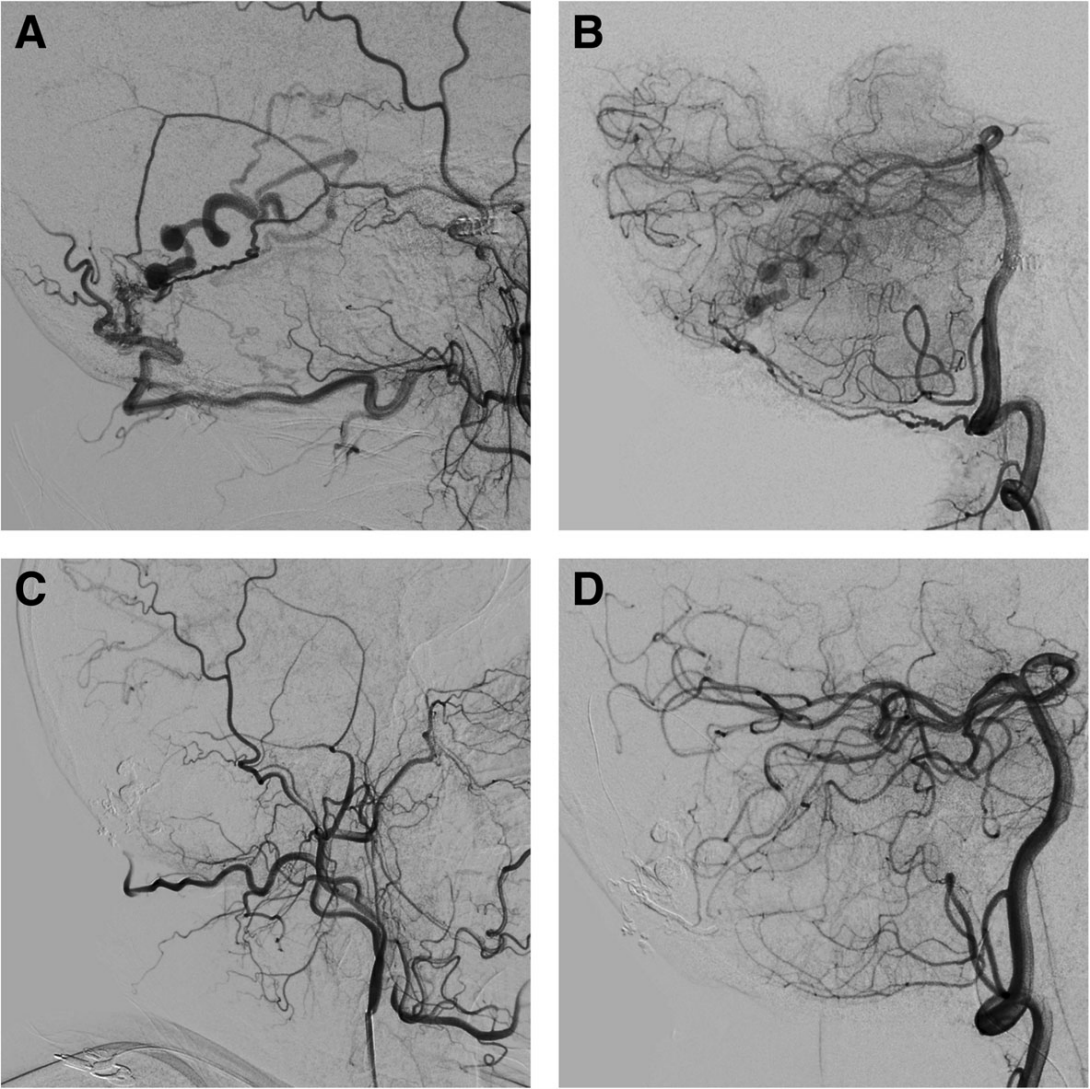
(**a**) Preoperative DSA of the right external carotid artery shows that the DAVF is fed by the branch of MMA and OA, and drains to the dilated occipital cortical vein. (**b**) DSA of the right vertebral artery shows that the DAVF also receives blood supply from the PMA. (**c**-**d**) DAVF disappears after Onyx embolization injection via the branch of MMA. DSA: digital subtraction angiography; DAVF: dural arteriovenous fistula; MMA: middle meningeal artery; OA: occipital artery; PMA: posterior meningeal artery

**Fig. 3:**
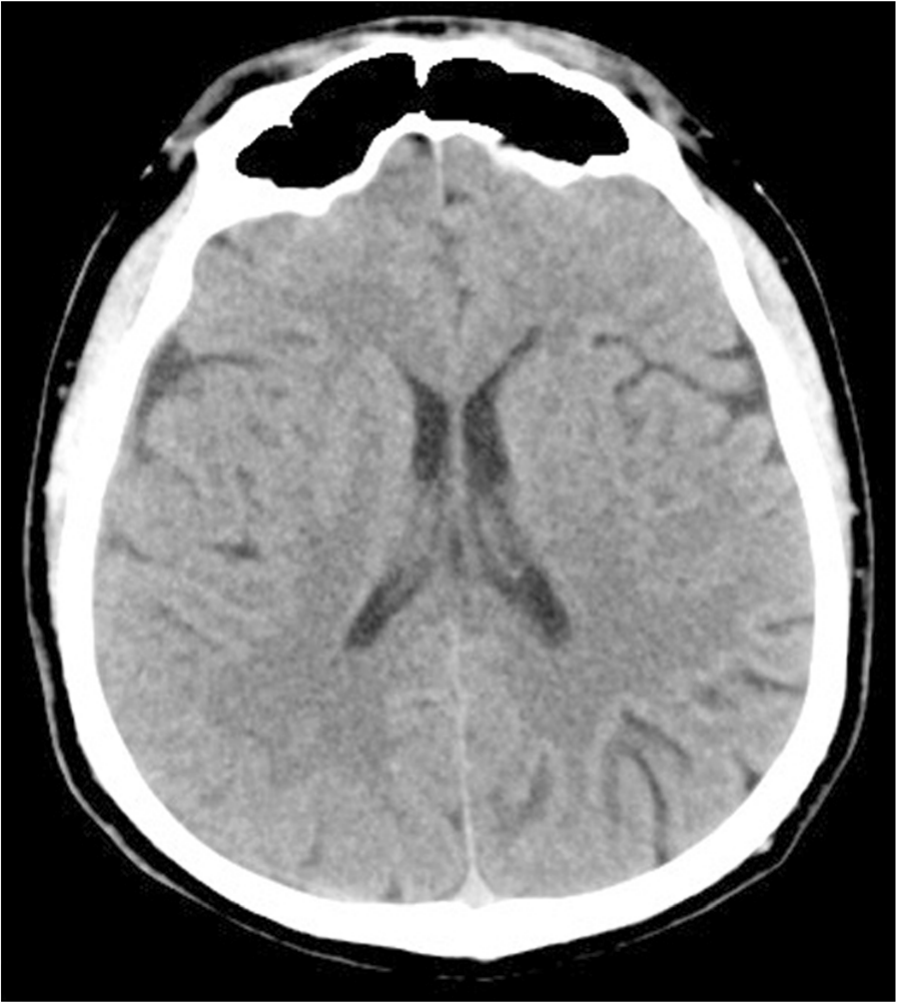
Head CT shows spontaneous resolution of the CSDH. CT: computed tomography; CSDH: chronic subdural hematoma

No anomaly was noticed during selective angiography of the internal carotid and vertebral arteries and the left external carotid artery. Selective angiography of the right external carotid artery showed that the DAVF was located at the transverse sinus and fed by posterior branch of the middle meningeal artery (MMA), the occipital artery, and the posterior meningeal artery and drained to the occipital cortical veins with venous ectasia (Fig. 2a-b). The DAVF was classified as type IV according to the Cognard classification. The embolization was performed via the MMA. The Headway duo catheter was used and accessed to the DAVF, and Onyx was injected until the shunt disappeared (Fig. 2c-d). The patient experienced an uneventful recovery. His CSDH gradually resolved in 1 month (Fig. 3). No neurologic deficit was noticed.

### Literature review

A PubMed search of published studies written in English and Chinese was conducted on June 30th, 2017. The following key words were used in relevant combinations: dural arteriovenous fistula, dural arteriovenous malformation, subdural hematoma, subdural haematoma, subdural hemorrhage, and subdural haemorrhage. The reference lists of the identified articles were also manually searched for additional studies. Studies of which full text could not be obtained or those without sufficient individualized description of the isolated SDH cases mixed in larger case series were excluded.

Finally, 13 articles containing 14 patients were identified [3–15]. In all 15 patients (9 females, 60%) including 1 case in our institution were included for the final interpretation (Table 1). The inflicted patients were aged from 27 to 82 years (55.5 ± 8.6).Of note, 12 (80%) of the 15 patients were aged between 40 and 60 years of age, and 8 (53.3%) patients were between 50 and 60 years. Sides of the DAVF or SDH were obtained in 13 patients with 9 (69.2%) located at the left side and 4 at the right side. The intracranial locations of DAVF were anterior fossa (2), middle fossa (2), frontal region (2), parietal region (3), temporal region (2), and occipital region (2). The types of SDH were CSDH (7/15), acute SDH (ASDH) (7/15), and undefined SDH (1/15). Of the 12 patients feeding artery could be identified, MMA was the commonest feeding artery (8/12, 66.7%). Multiple feeding arteries were identified in 3 (25%) patients. The causes of DAVF were only defined in 3 patients (2 iatrogenic and 1 traumatic), with the rest undefined or not mentioned. Cognard classifications (Table 2) of the DAVF were reported or deduced from the reports in 12 patients, with type I, type III, and type IV in 7, 2, and 3 patients respectively. The treatment strategies included hematoma evacuation (2/15), CSDH drainage and DAVF embolization (5/15), craniotomy and DAVF resection (3/15), DAVF embolization and hematoma evacuation (1/15), DAVF embolization (2/15), and not applicable or not mentioned (2/15). Of the 12 patients with direct description of outcome, 7 (58.3%) patients were neurological intact, 3 (25%) patients with neurological deficits, and 2 (16.7%) died.

**Table 1:** Clinical data of the patients with DAVF associated isolated SDH

Reference	Patient (Age, Sex)	Location of DAVF	Feeding artery	Cause of DAVF	Type of SDH	Cognard classification	Treatment	Outcome
Ito et al., 1983 [5]	64 years, M	Midline of the anterior fossa (R)	OPA	Undefined	ASDH	NA/NM	Hematoma evacuation with DAVF untreated	NA/NM
Halbach et al., 1988 [6]	48 years, F	Parietal (R)	Bilateral MMAs	Undefined	CSDH	NA/NM	CSDH drainage and direct MMA puncture and embolization	Complete resolution
Pappas CT et al., 1992 [7]	58 years, F	Parietal (L)	MMA	Iatrogenic	CSDH	Type I	Craniotomy and DAVF resection	Mild expressive aphasia
Başkaya MK et al., 1994 [8]	51 years, F	Anterior fossa (L)	AEA	Undefined	ASDH	Type I	Craniotomy and DAVF resection	Without Neurologic deficit
Komiyama M et al., 1994 [9]	58 years, F	Temporal (L)	MMA	Head Trauma	CSDH	Type I	DAVF embolization and burr-hole drainage	Without Neurologic deficit
Duffau H et al., 1999 [10]	55 years, M	NA/NM	NA/NM	NA/NM	SDH	Type III	DAVF embolization and Hematoma evacuation	Improved
	56 years, F	Middle Fossa	NA/NM	NA/NM	ASDH	Type III	Hematoma evacuation	Death
Maiuri F et al., 2001 [3]	59 years, F	Occipital (L)	MMA	Undefined	CSDH	TypeIV	NA/NM	NA/NM
Kominato et al., 2004 [11]	42 years, F	NA/NM (L)	NA/NM	Undefined	ASDH	NA/NM	NA/NM	Death
Kohyama S et al., 2009 [12]	60 years, M	Middle Fossa (L)	Bilateral MMAs	Undefined	ASDH	TypeI	DAVF embolization and burr-hole drainage	No neurological deficit
Ogawa K et al., 2010 [13]	27 years, M	Parietal (L)	OA	Undefined	ASDH	Type I	Hematoma evacuation and DAVF resection	No neurological deficit
de Aguiar GB et al., 2016 [14]	60 years, F	Frontal (R)	STA	Undefined	ASDH	TypeIV	DAVF embolization	Improved
Mewada T et al., 2016 [15]	82 years, F	Frontal (L)	MMA	Iatrogenic	CSDH	Type I	Burr-hole drainage and DAVF embolization	NA/NM
Kim E et al., 2016 [4]	67 years, M	Temporal (L)	MMA	Undefined	CSDH	Type I	Burr-hole drainage and DAVF embolization	Complete resolution
Present case	45 years, M	Occipital (R)	MMA,PMA, OA	Undefined	CSDH	TypeIV	DAVF embolization	Complete resolution

*M* male, *F*: female, *R* right, *L* left, *NA/NM* not applicable or not mentioned, *DAVF* dural arteriovenous fistula, *MMA* middle meningeal artery, *OPA* ophthalmic artery, *OA* occipital artery, *PMA* posterior meningeal artery, *AEA* anterior ethmoidal artery, *STA* superficial temporal artery, *SDH* subdural hematoma, *ASDH* acute subdural hematoma, *CSDH* chronic subdural hematoma

**Table 2:** Cognard classification of intracranial DAVF

Type	Venous drainage
Type I	Anterograde drainage into venous sinus
Type II	
IIA	Venous drainage into dural sinus with retrograde flow
IIB	Venous drainage into dural sinus with normal antegrade flow and CVD
IIA + B	Venous drainage into dural sinus with retrograde flow and CVD
Type III	Venous drainage directly into subarachnoid vein (CVD only)
Type IV	Venous drainage directly into subarachnoid vein with venous ectasia
Type V	Venous drainage directly into spinal perimedullar veins

*CVD* cortical venous drainage

## Discussion

DAVF is an uncommon subtype of intracranial AVMs [1]. In a Scottish population-based study in adults, the detection rate of DAVF was 0.16 per 100,000 adults per year, whereas the rate of all intracranial vascular malformations was 2.27 per 100,000 adults per year in the same population [16]. The manifestations of DAVF are diverse. In the recent Japanese survey by Kuwayama N et al., the initial clinical presentation was ocular symptoms, tinnitus, intracranial hemorrhage, and non-hemorrhagic neurological deficits in 45, 20, 16, and 20% of the patients respectively [2]. In the hemorrhagic patients, isolated SDH was only reported sporadically [3–15]. According to the literature, the majority of DAVFs are acquired in an idiopathic fashion, only a small proportion results from causes as trauma, infection, and iatrogenic injury [1, 17]. In this study, including our case, the causes of DAVF and associated SDH were only defined in 3 patients (2 iatrogenic and 1 traumatic), with the rest undefined or not mentioned.

The initial clinical presentation is not specific in patients with DAVF associated SDH. Just as presented in our case, headache (chronic, acute, or progressive) was the most common complaint. As a result of its rarity in occurrence, hardly could we ever associate DAVF with an SDH. In case of a patient presenting with SDH, there is no specific indication in imaging and clinical presentation that could imply the possibility of an underlying DAVF. However, there are some points that might indicate the existence of some underlying disorders: a) relatively young age, b) no evident history of head trauma, c) no coagulopathy or anticoagulation and antiplatelet medication, d) spontaneous occurrence of SDH, e) recurrent or refractory SDH that recurs in a short period after previous satisfactory hematoma evacuation.

The natural history of DAVF is primarily determined by the pattern of venous drainage [1, 17, 18]. Patients with cortical venous drainage (CVD) (especially with venous ectasia) have an aggressive natural history including ICH and nonhemorrhagic neurologic deficits (NHNDs). While DAVF without CVD manifests a benign process and rarely causes ICH or NHNDs. In this literature review, Cognard classification (Table 2) of the DAVF were reported or deduced from the reports in 12 patients, with type I, type III, and type IV in 7, 2, and 3 patients, respectively. Seven (58.3%) of the 12 patients harbored the supposed benign type I DAVF, which is somewhat inconsistent with the classical viewpoint [1, 17]. Hence, DAVF with CVD demonstrates an aggressive natural history and might be complicated with any kind of intracranial hemorrhage including SDH. The so-called benign type of DAVF without CVD does not always warrant a benign process and could also be complicated with SDH.

There was no consensus on the treatment of DAVF and its associated SDH. The treatment strategies depend on specific circumstances. In the case of massive ASDH, hematoma evacuation combined with simultaneous DAVF resection was the preferred strategy [7, 8, 13]. While burr-hole drainage combined with DAVF embolization was more suitable for CSDH patients [4, 6, 7, 12]. When the SDH did not cause evident intracranial hypertension or neurological deficit, mere DAVF embolization could also be selected [4, 14, 15]. Because the mass effect of CSDH in our patient was mild, we just embolized the DAVF at primary treatment. And the CSDH resolved spontaneously.

### Limitations

This is an isolated case report and a review of the literature. The data we interpreted were extracted from sporadic cases. As a result of the nature of this study, statistical analysis could not be conducted. Hence, the conclusion we achieved in the text is just a narrative interpretation of the past studies and future studies with larger case series are anticipated.

## Conclusion

DAVF is an uncommon subtype of intracranial AVMs. Isolated SDH including ASDH and CSDH is a rare complication of DAVF. The so-called benign type of DAVF without CVD does not always warrant a benign process and could also be complicated with SDH. Careful preoperative investigation is needed for relative young patients presenting with idiopathic or atypical SDH.

## Abbreviations

ASDH: Acute subdural hematoma
AVMs: Arteriovenous malformations
CSDH: Chronic subdural hematoma
CT: Computed tomography
CTA: CT angiography
CVD: Cortical venous drainage
DAVF: Dural arteriovenous fistula
DSA: Digital subtraction angiography
ICH: Intracerebral hemorrhage
MMA: Middle meningeal artery
NHNDs: Nonhemorrhagic neurologic deficits
SDH: Subdural hematoma
